# Characterizing LLM scientific concept generation: A multi-dimensional measurement study

**DOI:** 10.1371/journal.pone.0357892

**Published:** 2026-09-15

**Authors:** Jalil Ahmadpour

**Affiliations:** Independent Researcher, Tehran, Iran; Federal University of Paraiba, BRAZIL

## Abstract

Large Language Models (LLMs) can generate text describing scientific concepts, but the characteristics of these outputs remain poorly understood. We present a multi-dimensional characterization framework analyzing 9,666 outputs from seven models (GPT-4.1, GPT-5.2, GPT-5.5, o4-mini, Claude Sonnet 4.5, Claude Opus 4.5, and the open-weight Gemma-3-27B) across five scientific domains. Rather than making claims about creativity or novelty, we measure five independent dimensions: coherence, domain relevance, lexical profile, structural properties, and semantic position using four sentence embedding models spanning 2020–2024. After quality filtering (99.2% coherence, 99.9% domain relevance pass rates), we find that outputs exhibit graduate-level readability (median Flesch-Kincaid grade 16.3) and occupy semantic positions at the 83rd percentile of calibration distributions. All 39 metrics differ significantly across models (Kruskal-Wallis, *p* < 0.05), with structural properties showing the largest effects ($\epsilon^{2}$ = 0.35–0.54) and semantic position showing small-to-medium effects ($\epsilon^{2}$ ≈ 0.01–0.14). Dunn’s post-hoc tests with Bonferroni correction confirm that all model pairs differ on the top structural metrics (21/21 pairs for paragraph count, 20/21 for the next three). Centroid positions are robust to calibration sampling (bootstrap cosine similarity ≥ 0.99) and exceed a shuffled-domain baseline in 96.8–98.7% of cases; cross-model embedding consistency is moderate (Spearman ρ = 0.38–0.67) and is not explained by embedding dimensionality. Temperature effects replicate across two independent full-range models (GPT-4.1 and Gemma). This work provides calibrated measurements and validated methodology for future research without making interpretive claims about novelty.

## 1. Introduction

### 1.1. Motivation

Large Language Models have demonstrated remarkable capabilities in generating text across diverse domains, including descriptions of scientific concepts that may or may not correspond to established knowledge. Throughout this paper we use *scientific concept* to mean a self-contained proposition, mechanism, theory, or construct that purports to describe or explain a phenomenon within a scientific domain, and *scientific concept description* to mean the natural-language text a model produces when prompted to articulate such a concept; it is this text—not the validity of the underlying idea—that our framework measures. While prior work has explored LLM outputs in creative and scientific contexts [1–3], attempts to assess whether such outputs constitute genuine novelty rest on assumptions that have not been adequately validated—most critically, that semantic distance in embedding space serves as a reliable proxy for conceptual novelty. Recent human-subject work reports that LLM-generated research ideas can be rated by experts as more novel than human-generated ones. Yet that same work underscores how low inter-rater agreement on novelty is [1]. This motivates a shift away from adjudicating creativity and toward characterizing the measurable properties of model outputs.

### 1.2. The measurement problem

Embedding-based evaluation of text novelty relies on the assumption that greater distance from known concepts implies greater novelty. This assumption is problematic for several reasons. First, embedding models are optimized for semantic similarity, not novelty detection, and high distance may indicate incoherence, off-topic generation, or stylistic variation rather than conceptual innovation [4,5]. Second, different embedding models produce different distance distributions, making absolute thresholds arbitrary [6]. Third, single-metric dependency provides no convergent validity. These issues parallel broader concerns in computational creativity assessment about the gap between operational measures and theoretical constructs [7,8].

### 1.3. Research questions

We address three research questions:

**RQ1:** How do LLM-generated scientific concept descriptions differ across models on structural, lexical, and semantic dimensions?

**RQ2:** How do prompt framing and temperature settings affect output characteristics?

**RQ3:** How consistent are semantic position measurements across different embedding models?

These questions correspond to three expectations that connect the measurement framework to its objectives and that we evaluate descriptively rather than through inferential creativity claims. **H1** (linked to RQ1): because models differ in formatting, length, and verbosity, we expect cross-model differences to be *largest in structural properties* and comparatively smaller in domain-anchored semantic position. **H2** (linked to RQ2): we expect prompt framing and temperature to shift *surface* properties—lexical diversity, readability, and length—more than semantic position. **H3** (linked to RQ3): we expect semantic-position estimates to be only *moderately* consistent across embedding models, so that novelty claims resting on a single embedding model are unreliable. Each expectation is assessed through effect sizes (Sections 4.3–4.8) and the robustness analyses of Sections 4.9–4.11.

### 1.4. Approach and contributions

We propose a *characterization* approach rather than an *evaluation* approach, asking: “What are the measurable characteristics of LLM-generated scientific concept descriptions?” rather than making claims about creativity. Our contributions are: (1) a multi-dimensional characterization framework with 39 metrics across five dimensions; (2) coherence and domain relevance filtering methodology; (3) cross-model embedding validation showing moderate model dependency, supported by bootstrap centroid-stability and shuffled-domain baselines; and (4) calibrated measurements across seven models—including a frontier reasoning model and an open-weight model—five domains, three prompt types, and multiple temperature settings. Relative to existing multi-dimensional frameworks for LLM-generated ideas, which *score* idea quality or novelty (typically with LLM-judge panels or expert raters), our contribution is methodological: we characterize measurable textual properties directly and provide cross-model embedding-consistency evidence, centroid robustness and baseline validation, and a structural-versus-semantic comparison (Section 2.4).

## 2. Related work

### 2.1. LLM evaluation

Evaluation of LLM outputs spans a range of methodologies, from human evaluation [9] to automated benchmarks such as HELM [10] and MMLU [11]. For open-ended generation tasks, evaluation frameworks include both reference-based metrics (BLEU, ROUGE) and reference-free approaches using LLMs as judges [12]. However, evaluating *conceptual* generation—where the output purports to describe a scientific idea—poses unique challenges that standard methods do not address, as neither human evaluation at scale nor n-gram overlap metrics are suitable for assessing conceptual properties. A related but distinct line of work asks whether AI-generated scientific writing can be identified at all. Recent span-level detection methods combine section-conditioned stylistic modelling with contrastive learning, exploiting properties that extend beyond document-level semantic content [13]. That structural and stylistic signals suffice to localize machine-generated passages is independently informative here, because the present study finds structural properties to be the dimension along which model outputs differ most (Section 4.3). The aims differ—detection asks whether a text was machine generated, whereas we characterize how measurable properties vary across models and generation conditions—but both indicate that structure carries information that semantic content alone does not.

### 2.2. Computational creativity

The computational creativity literature provides theoretical frameworks for assessing creative outputs, including Boden’s distinction between combinational, exploratory, and transformational creativity [7], Colton’s creative tripod framework [14], and Wiggins’ formalization of creative search spaces [15]. The Consensual Assessment Technique [16] remains the gold standard for human creativity evaluation. Recent work has applied creativity frameworks to LLM outputs [2,3,17], but the gap between theoretical creativity constructs and operationalizable measurements remains substantial. This gap is not peculiar to computational settings. Bibliometric mapping of the wider creativity literature describes a heterogeneous, networked field in which distinct intellectual streams conceptualize creativity differently [18]. That creativity is poorly served by any single observable indicator is the same consideration that motivates our multi-dimensional treatment of model outputs. Our work sidesteps this gap by providing empirical measurements rather than creativity claims.

### 2.3. Sentence embeddings

Sentence embedding models have become standard tools for measuring semantic similarity [19,20]. The evolution from models like Sentence-BERT [19] and all-mpnet-base-v2 to modern architectures including BGE [21], Nomic Embed [22], and GTE [23] has improved representation quality but also introduced model-dependent variation in distance distributions. Prior work on embedding-based novelty detection in scientific text [24] has typically relied on a single model, limiting the robustness of findings. Our use of four models spanning 2020–2024 directly addresses this limitation. Because learned representation spaces are shaped by architecture, dimensionality, and training objective, semantic distance should not be treated as an architecture-independent quantity; Section 4.7 quantifies how far the choice of embedding model shifts semantic-position estimates in practice.

### 2.4. LLM concept generation

Several studies have explored LLM capabilities in scientific ideation and concept generation [1,25,26]. These typically assess output quality through expert evaluation or embedding distance from known concepts. Closest to our setting, recent benchmarks characterize idea generation across many scientific domains along multiple dimensions. One such effort evaluates divergent-thinking quality across 22 domains using a panel of LLM judges [27], and another shows that reference-grounded scoring captures quality dimensions that generic similarity scores miss [28]. We differ from both in computing structural, lexical, coherence, and semantic-positioning metrics *directly* on the text rather than relying on LLM-judge or reference-grounded scores. A recent survey of LLM scientific ideation [29] organizes this fast-growing area using established creativity frameworks and a taxonomy of generation methods, and confirms that its evaluation effort is oriented overwhelmingly toward *judging* the novelty and quality of generated ideas. Our study is deliberately orthogonal to that effort: rather than scoring idea quality, we characterize the measurable textual properties of model outputs and ask whether the embedding-distance signals such judgments often rely on are themselves stable and meaningful. What is genuinely novel here is therefore not another quality benchmark but (i) a cross-model embedding-consistency analysis showing that single-embedding semantic-position estimates are only moderately reliable; (ii) bootstrap and shuffled-domain validation establishing that domain centroids are stable and carry genuine domain signal; and (iii) the finding that, under the semantic-position operationalization adopted here, cross-model structural variation is several-fold larger than semantic-distance variation, which suggests that embedding distances read as “novelty” in some prior work substantially reflect style and structure. A common limitation of prior work is the conflation of embedding distance with novelty without controlling for text quality, style, or domain relevance. Our multi-dimensional approach addresses these confounds by measuring structural and lexical properties alongside semantic position, enabling researchers to disentangle stylistic variation from semantic distance.

## 3. Methodology

### 3.1. Data collection

#### 3.1.1. Models.

We collected outputs from seven state-of-the-art LLMs representing standard, reasoning-oriented, and open-weight architectures (Table 1). Five models (GPT-4.1, GPT-5.2, o4-mini, Claude Sonnet 4.5, Claude Opus 4.5) were generated in the original study (January 2026). Two further models—GPT-5.5, a frontier reasoning model, and Gemma-3-27B-Instruct, an open-weight model run locally with quantization-aware-training weights—were added during revision (June 2026) under the identical protocol. Standard models support explicit temperature control; reasoning models (GPT-5.2, o4-mini, GPT-5.5) do not expose a temperature parameter and were run at their default settings. The five-month gap between the original and added generations reflects the revision timeline; because all comparisons are descriptive and within a fixed protocol, we do not expect it to bias the cross-model characterization, and we note it here for transparency. The complete set of generated outputs and the analysis code used in this study are archived at Zenodo [30].

**Table 1:** Models used for generation.

Model	Type	Vendor	Records	Temperature
GPT-4.1	Standard	OpenAI	2,219	0.7, 1.0, 1.3
Claude Sonnet 4.5	Standard	Anthropic	1,498	0.7, 1.0
Claude Opus 4.5	Standard	Anthropic	1,487	0.7, 1.0
GPT-5.2	Reasoning	OpenAI	750	Default only
o4-mini	Reasoning	OpenAI	749	Default only
GPT-5.5	Reasoning	OpenAI	713	Default only
Gemma-3-27B (QAT)	Open-weight	Google	2,250	0.7, 1.0, 1.3

Record counts reflect totals after coherence and domain relevance filtering (Section 3.2). The unbalanced temperature design reflects genuine API constraints: Claude models and the open-weight Gemma model were tested at multiple temperatures, GPT-4.1 and Gemma at the full three-temperature range, and reasoning models at their default only. The addition of Gemma provides a second model spanning the full temperature range (Section 4.5), allowing the temperature trends to be checked for replication independently of GPT-4.1.

#### 3.1.2. Domains and calibration.

Five scientific domains were selected to span distinct epistemic styles within a tractable set: physics, biology, mathematics, philosophy, and economics. These cover the empirical natural sciences (physics, heavily mathematical; biology, descriptive and mechanism-driven), the formal sciences (mathematics, where claims are proof-based and notation-heavy), the humanities (philosophy, where reasoning is discursive rather than empirical), and the social sciences (economics, which blends formal modeling with empirical and theoretical argument). This spread deliberately exercises the framework against domains that differ markedly in vocabulary, symbolic content, and rhetorical structure—the conditions under which domain-dependent measurement artifacts are most likely to surface (for example, syllable-based metrics under-counting mathematical notation; Section 4.6).

Each domain was represented by 31 calibration concepts, selected to span its range of establishment rather than to sample it exhaustively: 20 *control* concepts (canonical, textbook-level ideas central to the domain), 6 *known* concepts (established but more specialized topics with a dedicated literature), and 5 *novel* concepts (recent or speculative proposals at the field’s frontier), totaling 155 calibration concepts. This three-tier composition is intended to anchor each domain centroid in its consensus core while still representing its periphery, so that an output’s distance reflects its position relative to the domain’s spread rather than to a single canonical point. Expanded descriptions of 300–500 tokens per concept were used to improve centroid estimation. Because 31 concepts is a modest basis for estimating a centroid in 768–1024-dimensional space, we do not treat any single centroid as ground truth; the sensitivity of the centroids to this selection is quantified directly by bootstrap resampling (Section 4.9), where recomputed centroids remain within cosine similarity 0.990–0.999 of the full-set centroid across all domains and embedding models.

#### 3.1.3. Prompt design.

Three prompt types were designed to elicit concept generation through different framings:

**Direct invention:** Instructs the model to invent or create a new scientific concept within the target domain.

**Discovery framing:** Instructs the model to describe a concept as if it were a genuine scientific discovery.

**Theoretical framework:** Instructs the model to propose a theoretical framework addressing an open problem in the domain.

Each combination of model, domain, prompt type, and temperature yielded 50 independent generations. The full prompt texts are provided in supplementary materials. The original generation run spanned January 3–6, 2026, producing 6,770 raw outputs; the two models added in revision were generated in June 2026 under the identical protocol, producing a further 3,000 raw outputs (GPT-5.5: 750; Gemma-3-27B: 2,250), for 9,770 raw outputs in total.

### 3.2. Quality filtering

**Fig 1:**
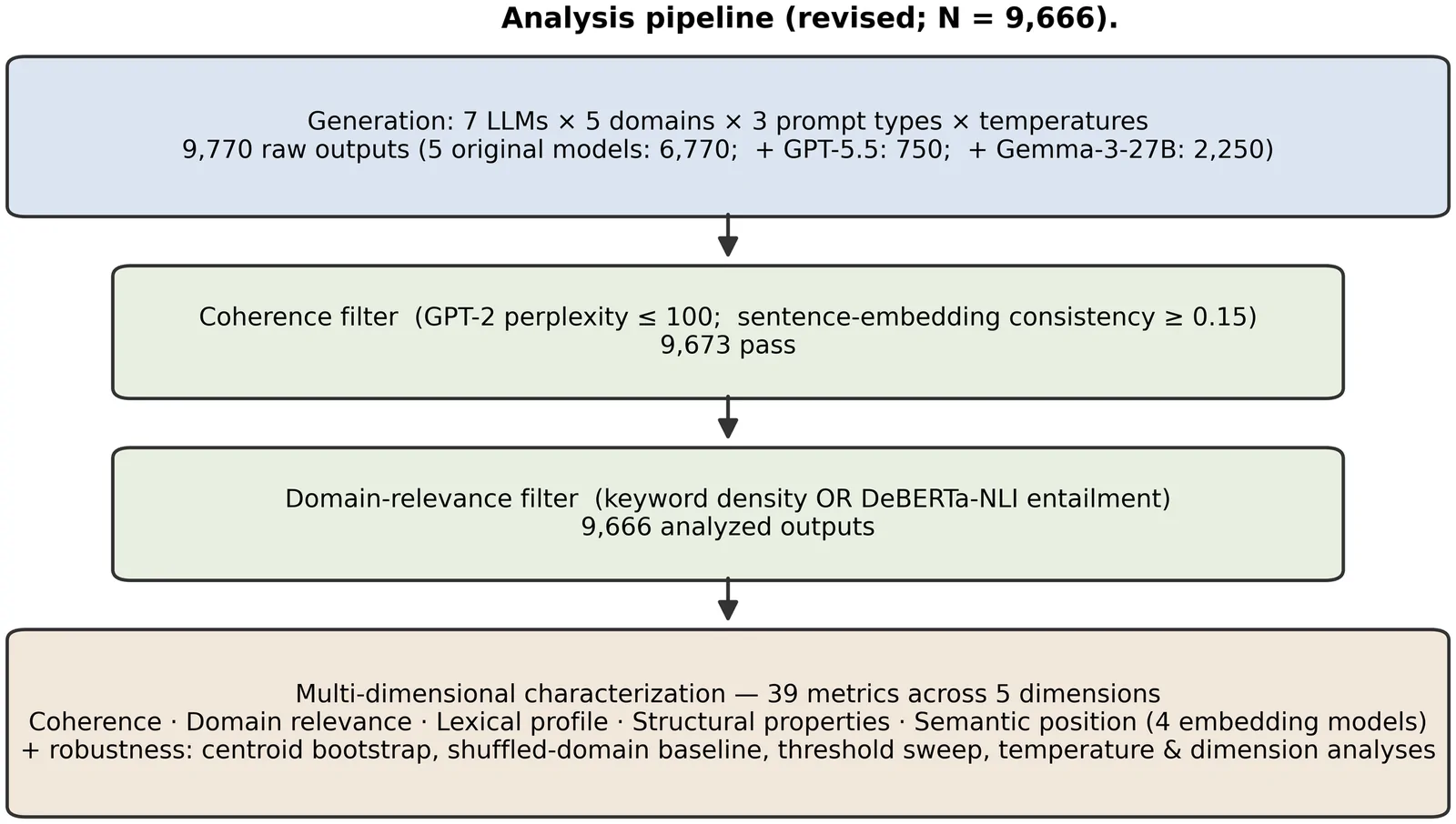
Analysis pipeline overview (revised; N = 9,666). Records are filtered for coherence and domain relevance before multi-dimensional characterization.

Fig 1 illustrates the analysis pipeline.

#### 3.2.1. Coherence filtering.

All outputs were filtered using two coherence metrics. **Perplexity** was computed using GPT-2 [31], with outputs exceeding 100 flagged as incoherent. **Semantic consistency** was measured as mean pairwise cosine similarity of sentence embeddings (all-MiniLM-L6-v2), with outputs below 0.15 flagged. The semantic consistency threshold was set at 0.15 rather than the initially planned 0.30 because pilot analysis revealed that long, multi-topic scientific descriptions naturally exhibit lower inter-sentence similarity; a threshold of 0.30 rejected 69% of outputs including visually coherent text. We report results across the full range of thresholds in Section 4.10. Grammar checking via LanguageTool was attempted but excluded as a filter because the tool failed to parse the majority of outputs (markdown, mathematical notation, and long technical prose triggered systematic Java parsing failures; 96% error rate); to verify that this exclusion does not admit ungrammatical text, we instead conducted a manual grammatical audit of a stratified sample of 200 outputs (Section 4.12, Table D in S1 File). Of 9,770 raw generations, 20 were empty or failed and 9,673 passed coherence filtering (99.2% pass rate).

#### 3.2.2. Domain relevance filtering.

Coherent outputs were filtered using two methods: keyword density (curated domain-specific term lists, 30–50 terms per domain) and zero-shot classification (DeBERTa-v3-base fine-tuned on NLI [32]). An output passed if either its keyword density exceeded 0.5% or the classifier’s entailment score exceeded 0.3. Of 9,673 coherent outputs, 9,666 passed domain relevance filtering (99.9% pass rate); the final analyzed corpus comprises 9,666 outputs. The original five models reproduced their published counts exactly (6,703 analyzed records), confirming that the two added models are strictly additive.

### 3.3. Multi-dimensional characterization

Each filtered output was characterized across five dimensions comprising 39 metrics. Complete definitions are given in Table A of S1 File.

**Dimension 1—Coherence:** Perplexity (GPT-2) and semantic consistency (sentence embedding similarity). Retained from filtering as continuous metrics.

**Dimension 2—Domain Relevance:** Domain keyword density and core keyword hit count.

**Dimension 3—Lexical Profile:** Six metrics: type-token ratio (lexical diversity), out-of-vocabulary rate (relative to NLTK English dictionary), vocabulary overlap with domain calibration descriptions (shared tokens), technical term density (proportion of polysyllabic words), hapax legomena ratio (words appearing exactly once), and average syllables per word.

**Dimension 4—Structural Properties:** Six metrics: average sentence length, sentence count, paragraph count, Flesch-Kincaid grade level, Flesch reading ease score, and definition structure density.

**Dimension 5—Semantic Position:** For each of four embedding models: cosine and Euclidean distance to domain centroid, nearest calibration concept distance, average distance to all calibration concepts, percentile rank in calibration distribution, and an in-distribution flag (within 2σ). Cross-model consistency metrics: mean, standard deviation, and range of percentile ranks across models. For each embedding model, the domain centroid is the mean of the L2-normalized calibration embeddings:


$$\begin{array}{c} {𝐜}_{d}=\frac{1}{\left|C_{d}\right|}\sum_{i\in C_{d}}{\hat{𝐞}}_{i},\;{\hat{𝐞}}_{i}=\frac{{𝐞}_{i}}{\left\|{𝐞}_{i}\right\|} \end{array}$$

and the cosine distance of an output embedding 𝐞 to its domain centroid is


$$\begin{array}{c} d_{\cos}(𝐞,{𝐜}_{d})=1-\frac{𝐞\cdot {𝐜}_{d}}{\left\|𝐞\|\;\|{𝐜}_{d}\right\|} \end{array}$$

with the Euclidean variant using $\left\|\hat{𝐞}-{𝐜}_{d}\right\|$. Percentile rank is the output’s rank within the distribution of calibration-concept distances for that domain, and the in-distribution flag marks outputs within 2σ of the calibration mean distance.

This construction targets *semantic position*—where an output sits relative to a domain’s established vocabulary of meaning—rather than its length or verbosity. Two features make the separation explicit. First, embeddings are L2-normalized before centroids and distances are computed, so cosine distance is invariant to output length and overall magnitude; verbosity by itself does not move an output toward or away from a centroid. Second, length and structure are measured *independently* as Dimension-4 metrics (sentence count, paragraph count, sentence length), so any length signal is captured there rather than confounded into the semantic-position metrics. The decisive check is empirical: if centroid distance reflected verbosity rather than domain meaning, outputs would not be systematically closer to their *own* domain centroid than to other domains’ centroids—yet they are, in 96.8–98.7% of cases relative to wrong-domain and label-permuted baselines (Section 4.9). Length alone cannot produce domain-specific separation of this kind.

### 3.4. Embedding models

Semantic position was computed in four sentence-embedding spaces spanning 2020–2024. Table 2 lists these models with their identifiers, embedding dimensionalities, and sources.

**Table 2:** Sentence embedding models for semantic position analysis.

Name	Year	Model ID	Dims	Source
mpnet-2020	2020	all-mpnet-base-v2	768	Sentence-Transformers [19]
bge-2023	2023	bge-large-en-v1.5	1024	BAAI [21]
nomic-2024	2024	nomic-embed-text-v1.5	768	Nomic AI [22]
gte-2024	2024	gte-large-en-v1.5	1024	Alibaba NLP [23]

### 3.5. Statistical approach

All analyses are descriptive. Cross-condition comparisons use the Kruskal-Wallis *H* test [33], a non-parametric rank-based test for comparing distributions across groups. We report effect sizes as epsilon-squared ($\epsilon^{2}$ = *H* / (*N* – 1)), interpreted using conventional benchmarks as negligible (< 0.01), small (0.01–0.06), medium (0.06–0.14), or large (≥ 0.14) [34]. Post-hoc pairwise comparisons use Dunn’s test with Bonferroni correction [35]. We emphasize effect magnitudes over *p*-values, as the large sample size (*N* = 9,666) provides power to detect trivially small effects.

Perplexity is computed with GPT-2 as a deliberately fixed, lightweight, and reproducible reference language model for relative coherence screening rather than as a measure of fluency quality; because GPT-2 is far smaller and older than the systems under study, it provides a neutral common yardstick and avoids the circularity that would arise from scoring a model with a member of its own family. We chose GPT-2 specifically—rather than a newer open model such as Llama or Mistral—for three reasons: it is small and deterministic enough to score tens of thousands of outputs cheaply and reproducibly; its 2019 training era predates every evaluated system, so it cannot have been tuned on their outputs; and its modest capacity makes it a deliberately *neutral* yardstick unlikely to share idiosyncrasies with any single evaluated family. A newer reference would reintroduce the circularity we wish to avoid—scoring a model with a near-sibling—without changing the relative ordering our screening uses. We emphasize that the resulting values are not pure measures of output fluency: a substantial part of their *absolute* level reflects the mismatch between GPT-2’s 2019 distribution and the text produced by far larger modern models, so a fluent modern output can still receive a high GPT-2 perplexity. For this reason absolute perplexity values are scale-dependent and are used only comparatively—for coherence screening at a permissive ≤ 100 threshold and for within-condition comparison—never as an absolute quality score, and we read cross-model perplexity differences as reflecting distributional distance from GPT-2 as much as differences in intrinsic fluency. Likewise, the embedding calibration provides a fixed reference frame, not ground truth.

### 3.6. Ethics statement

This study involves no human participants. All data was generated by commercial LLM APIs and one locally run open-weight model. We note that characterization of LLM concept generation capabilities has potential dual-use implications: the same measurements that enable scientific understanding of model outputs could theoretically inform automated generation of misleading scientific claims. We mitigate this concern by focusing on characterization methodology rather than optimization of novelty-appearing outputs.

## 4. Results

We organize the results to separate the study’s *primary methodological contributions* from *supporting robustness analyses*. The primary results are the multi-dimensional characterization and the three research questions: the overall metric profile (Section 4.2), cross-model variation (Section 4.3, RQ1), prompt and temperature effects (Sections 4.4–4.5, RQ2), and cross-embedding consistency (Section 4.7, RQ3). The analyses that follow these—bootstrap centroid stability and the shuffled-domain baseline (Section 4.9), threshold sensitivity (Section 4.10), embedding-architecture controls (Section 4.11), and the manual grammar audit (Section 4.12)—are supporting analyses that validate the robustness of the primary findings rather than introduce new claims.

### 4.1. Data overview

Of 9,770 raw generations, 20 were empty or failed, 9,673 passed coherence filtering (99.2%), and 9,666 passed domain relevance filtering (99.9%). The high pass rates indicate that current LLMs consistently produce coherent, domain-relevant text when prompted to generate scientific concepts, and they hold across all seven models including the open-weight Gemma model.

### 4.2. Overall characterization

Table 3 reports the overall distribution of the key characterization metrics. Outputs exhibit graduate-level readability (median Flesch-Kincaid grade 16.3), moderate lexical diversity (type-token ratio 0.496), and a 20.7% out-of-vocabulary rate reflecting heavy use of technical terminology. In semantic position, outputs occupy the outer portions of calibration distributions, with a cross-model mean percentile of 83.1. The distance scale varies substantially across embedding models: mpnet-2020 produces median cosine distances of 0.522 while newer models cluster around 0.20–0.27, reflecting differences in embedding space geometry.

**Table 3:** Overall distribution of key characterization metrics (N = 9,666).

Metric	Median	Mean	SD	P5	P95
Perplexity (GPT-2)	35.2	36.8	13.1	17.5	61.1
Semantic consistency	0.270	0.274	0.057	0.187	0.372
Type-token ratio	0.496	0.499	0.102	0.328	0.675
OOV rate	0.207	0.206	0.044	0.128	0.280
Technical term density	0.337	0.333	0.064	0.217	0.433
Hapax legomena ratio	0.683	0.684	0.065	0.578	0.796
Vocabulary overlap	0.569	0.568	0.062	0.466	0.668
Avg sentence length (words)	14.3	14.4	4.5	7.4	22.0
Flesch-Kincaid grade	16.3	16.1	3.4	9.9	21.5
Flesch reading ease	4.3	5.6	18.7	−23.0	41.2
Centroid dist. (mpnet-2020)	0.522	0.530	0.099	0.376	0.705
Centroid dist. (bge-2023)	0.219	0.222	0.029	0.182	0.275
Centroid dist. (nomic-2024)	0.201	0.207	0.033	0.162	0.271
Centroid dist. (gte-2024)	0.271	0.278	0.042	0.221	0.360
Cross-model percentile mean	83.1	81.9	11.6	61.3	98.4

### 4.3. Variation by model (RQ1)

All 39 metrics show statistically significant differences across models (*p* < 0.05 for all). Effect sizes (Table 4) reveal that the largest cross-model differences are in structural properties: paragraph count ($\epsilon^{2}$ = 0.54, large), average sentence length ($\epsilon^{2}$ = 0.47, large), sentence count ($\epsilon^{2}$ = 0.38, large), and semantic consistency ($\epsilon^{2}$ = 0.35, large). Semantic-position metrics show predominantly small-to-medium effects ($\epsilon^{2}$ ≈ 0.01–0.14). Adding the two new models leaves this ordering unchanged and, if anything, sharpens it. This ordering is conditional on the semantic-position operationalization adopted here—centroid distances in four embedding spaces, calibrated as in Section 3.1.2; under a different semantic representation the size of the gap could differ, although the structural effects are large in absolute terms regardless.

**Table 4:** Median metric values by model with effect sizes.

Metric	C.Opus	C.Sonnet	GPT-4.1	GPT-5.2	GPT-5.5	o4-mini	Gemma-3-27B	$\epsilon^{2}$	Size
Paragraph count	49	27	20	46	123	8	16	0.54	Large
Avg sentence length	11.7	12.3	16.6	8.0	9.8	13.7	17.8	0.47	Large
Sentence count	91	54	35	83	167	39	54	0.38	Large
Semantic consistency	0.236	0.266	0.320	0.240	0.218	0.249	0.288	0.35	Large
FK grade	15.1	16.5	17.3	13.7	12.5	15.1	17.5	0.26	Large
Type-token ratio	0.458	0.542	0.541	0.508	0.358	0.570	0.469	0.24	Large
Technical density	0.328	0.361	0.342	0.328	0.279	0.299	0.350	0.15	Large
Centroid dist. (gte)	0.262	0.263	0.289	0.249	0.259	0.292	0.272	0.09	Med.
Centroid dist. (mpnet)	0.516	0.515	0.546	0.493	0.490	0.527	0.524	0.02	Small
Cross-model pct. mean	82.3	82.3	83.1	76.6	79.8	85.5	87.9	0.04	Small

**Fig 2:**
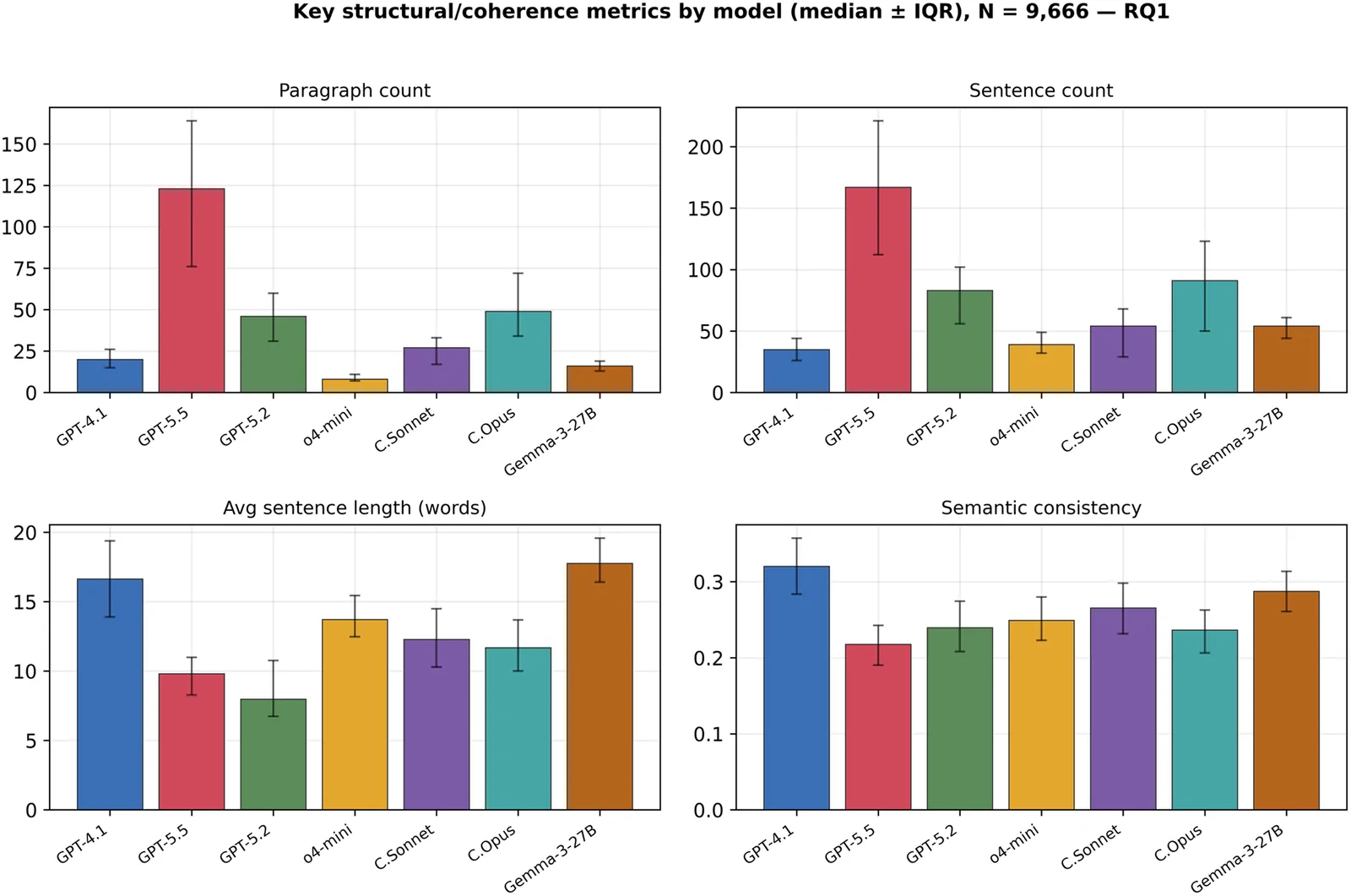
Key structural/coherence metrics by model (median ± IQR), N = 9,666. Structural metrics show the largest cross-model variation.

The two added models occupy distinct structural positions (Fig 2). GPT-5.5 is a pronounced outlier, producing by far the most segmented outputs (median 123 paragraphs and 167 sentences, with short sentences of 9.8 words), whereas Gemma-3-27B is the most prose-like model, with the fewest paragraphs among the long generators (16) and the longest sentences (17.8 words). Among the original models, Claude Opus remains highly extensive (91 sentences, 49 paragraphs) and o4-mini the most compact (8 paragraphs); GPT-4.1 retains the highest semantic consistency (0.320). The structural effect sizes (0.35–0.54) remain several-fold to roughly an order of magnitude larger than semantic-position effects (mostly 0.02–0.13), indicating that models differ far more in how they organize text than in what semantic territory they occupy.

Dunn’s post-hoc tests with Bonferroni correction confirm that these differences are not driven by a single outlier model. With seven models there are 21 pairwise comparisons. For paragraph count ($\epsilon^{2}$ = 0.54), all 21 pairs are statistically significant (*p* < 0.05 adjusted). For average sentence length, sentence count, and semantic consistency, 20 of 21 pairs are significant in each case, and for Flesch-Kincaid grade 20 of 21. These results demonstrate that the cross-model variation reflects genuine systematic differences across the full set of seven models, not merely one model deviating from the rest.

### 4.4. Variation by prompt type (RQ2)

**Fig 3:**
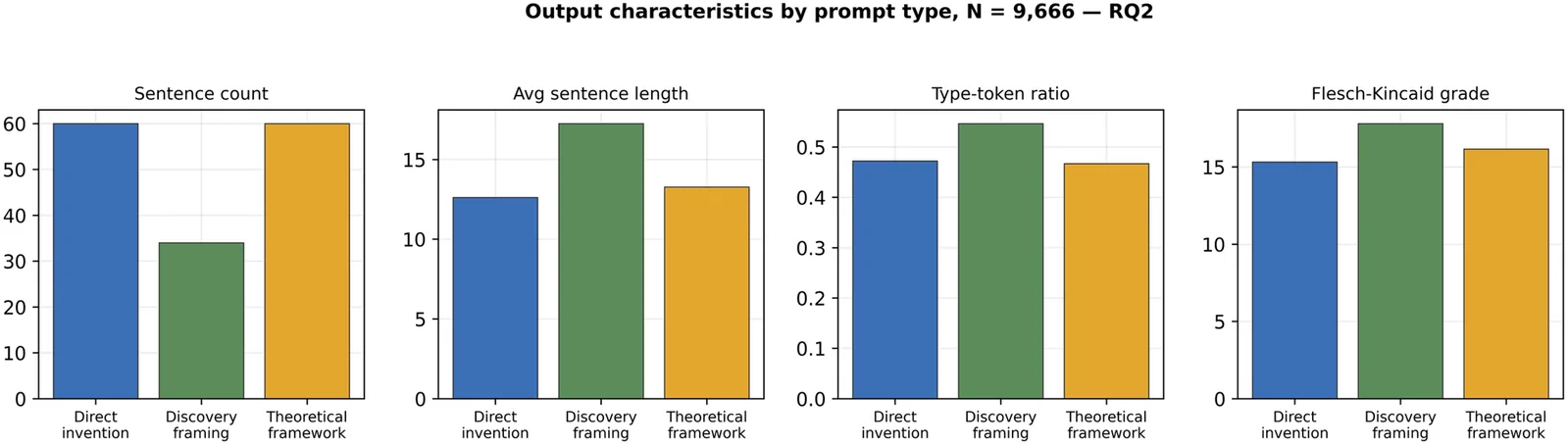
Output characteristics by prompt type, N = 9,666. Discovery framing produces more academic, prose-like text; direct invention produces the most semantically distant outputs.

Discovery framing produces the most distinctive outputs (Fig 3): fewer but longer sentences (34 sentences at 17.3 words each), higher lexical diversity (TTR 0.547), and notably smaller semantic distances (cross-model percentile 78.2). This pattern is consistent with discovery framing eliciting more academic, prose-like writing that stays closer to established concept descriptions. Direct invention framing produces semantically more distant outputs (percentile 86.3). Eight of the 35 tested metrics show large prompt-type effects ($\epsilon^{2}$ ≥ 0.14; Table 5). Dunn’s post-hoc tests show all three pairwise comparisons are significant for the top metrics (sentence count, centroid distance, TTR), except for hapax legomena ratio where direct invention and theoretical framework do not differ significantly (*p* = 1.0 adjusted). The exception aside, these tests confirm that discovery framing is the primary driver of prompt-type variation.

**Table 5:** Median values by prompt type with effect sizes.

Metric	Direct Invention	Discovery	Theoretical FW	$\epsilon^{2}$	Size
Sentence count	60	34	60	0.23	Large
Centroid dist. (nomic)	0.219	0.188	0.202	0.19	Large
Hapax legomena ratio	0.664	0.717	0.664	0.18	Large
Type-token ratio	0.472	0.547	0.467	0.17	Large
FK grade	15.3	17.8	16.2	0.16	Large
Avg sentence length	12.6	17.3	13.3	0.15	Large
Paragraph count	24	16	28	0.04	Small
Cross-model pct. mean	86.3	78.2	83.9	0.06	Med.

### 4.5. Temperature effects (RQ2)

**Fig 4:**
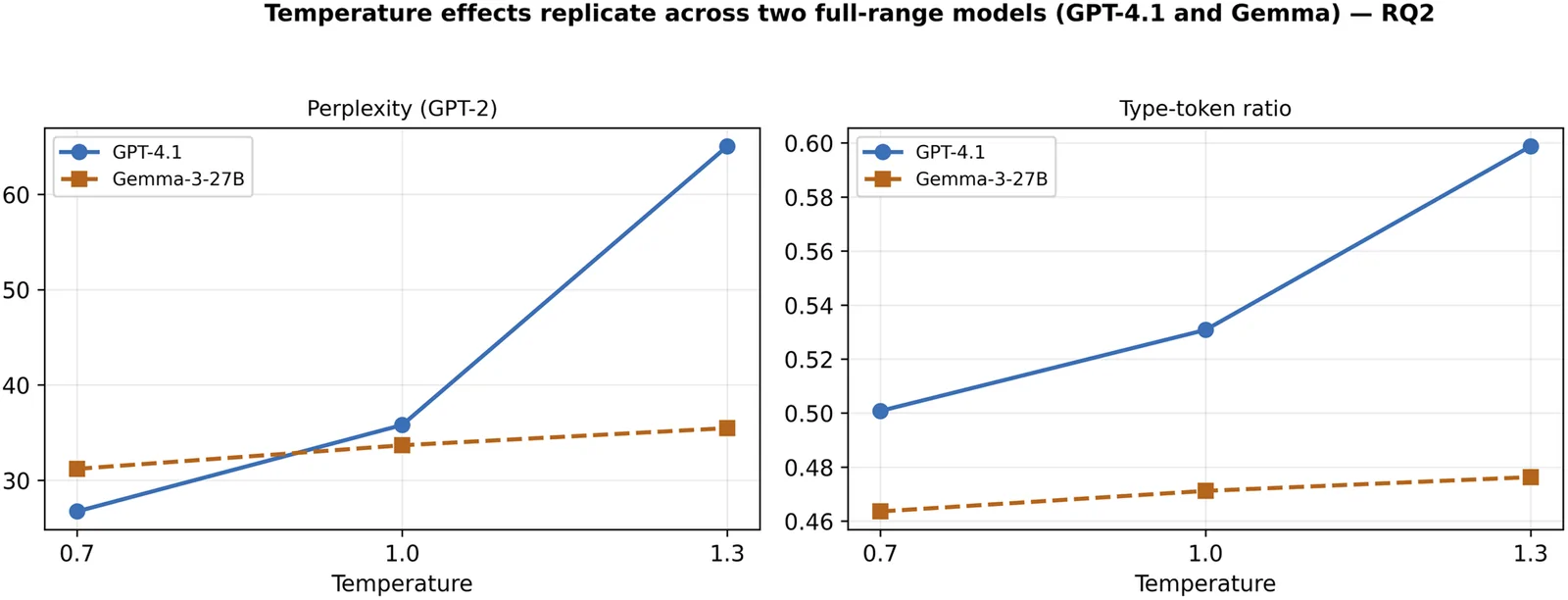
Temperature effects replicate across two independent full-range models (GPT-4.1 and Gemma). Higher temperature produces monotonically more complex, diverse, and perplexing text.

Two models—GPT-4.1 and the open-weight Gemma-3-27B—provide the full three-temperature range, allowing the temperature trends to be checked for replication across vendors and architectures (Fig 4). Claude models contribute two temperatures and reasoning models their default only, so the pooled temperature design remains unbalanced across the full model set.

Temperature produces largely monotonic effects in the pooled data (Table 6). Perplexity rises from 31.8 at t = 0.7 to 42.6 at t = 1.3, readability becomes more complex (Flesch reading ease drops from 3.9 to −3.0), and lexical diversity increases (TTR from 0.484 to 0.524). The pooled t = 1.3 perplexity (42.6) is lower than in the original five-model analysis because the open-weight Gemma model—whose perplexity rises more gently with temperature—is now included; the within-model trend for GPT-4.1 is unchanged (Section 4.5.1, Fig 4). Higher temperature produces shorter outputs, but with longer, more complex sentences and more unique vocabulary. This is consistent with the generation process sampling from lower-probability tokens. Post-hoc tests confirm that all six pairwise temperature comparisons are significant for perplexity and Flesch reading ease; five of six are significant for average sentence length, Flesch-Kincaid grade, semantic consistency, and technical-term density.

**Table 6:** Median values by temperature with effect sizes (pooled across models).

Metric	t = 0.7	t = 1.0	t = 1.3	Default	$\epsilon^{2}$	Size
Perplexity	31.8	36.0	42.6	35.3	0.10	Med.
Hapax ratio	0.672	0.681	0.709	0.692	0.04	Small
FK grade	16.4	16.7	18.2	13.7	0.21	Large
Avg sent. length	14.3	14.7	18.1	10.6	0.23	Large
FRE	3.9	2.4	−3.0	15.9	0.15	Large
TTR	0.484	0.498	0.524	0.497	0.05	Small
Sem. consistency	0.278	0.275	0.297	0.235	0.13	Med.
Sentence count	52	51	44	71	0.07	Med.

#### 4.5.1. Within-model temperature effects.

To isolate temperature from model identity, we computed Spearman correlations between temperature and each metric *within GPT-4.1* (n = 2,219), the model with the most balanced temperature coverage. Temperature correlates strongly with perplexity (ρ = 0.77) and with lexical-variety measures (hapax legomena ratio ρ = 0.52, type-token ratio ρ = 0.44; Flesch-Kincaid grade ρ = 0.37; Flesch reading ease ρ = −0.35). By contrast, the correlation with technical-term density is weak (ρ = 0.20). The independent open-weight model, Gemma-3-27B, reproduces the same directional pattern across its three temperatures (Fig 4). Perplexity (31.2 → 33.7 → 35.5), type-token ratio (0.464 → 0.471 → 0.476), technical-term density (0.346 → 0.348 → 0.353), and average sentence length (17.5 → 17.8 → 18.0) all increase monotonically from 0.7 to 1.3. Together these results indicate that higher temperature increases **surface lexical variability**—rarer tokens, more unique words, longer and more complex sentences—without a commensurate change in **technical substance**, consistent with sampling from lower-probability tokens.

### 4.6. Variation by domain

Domain differences are evident across several dimensions. Biology shows the highest technical term density (median 0.399), followed by economics (0.365) and physics (0.333). Mathematics shows the lowest technical term density (0.288) and the lowest type-token ratio (0.432), reflecting both its reliance on symbolic notation not captured by syllable-based measures and its more repetitive use of formal terminology. Physics and mathematics show the largest centroid distances on most embedding models, while economics and philosophy sit closer to their domain centroids.

### 4.7. Cross-model embedding consistency (RQ3)

**Fig 5:**
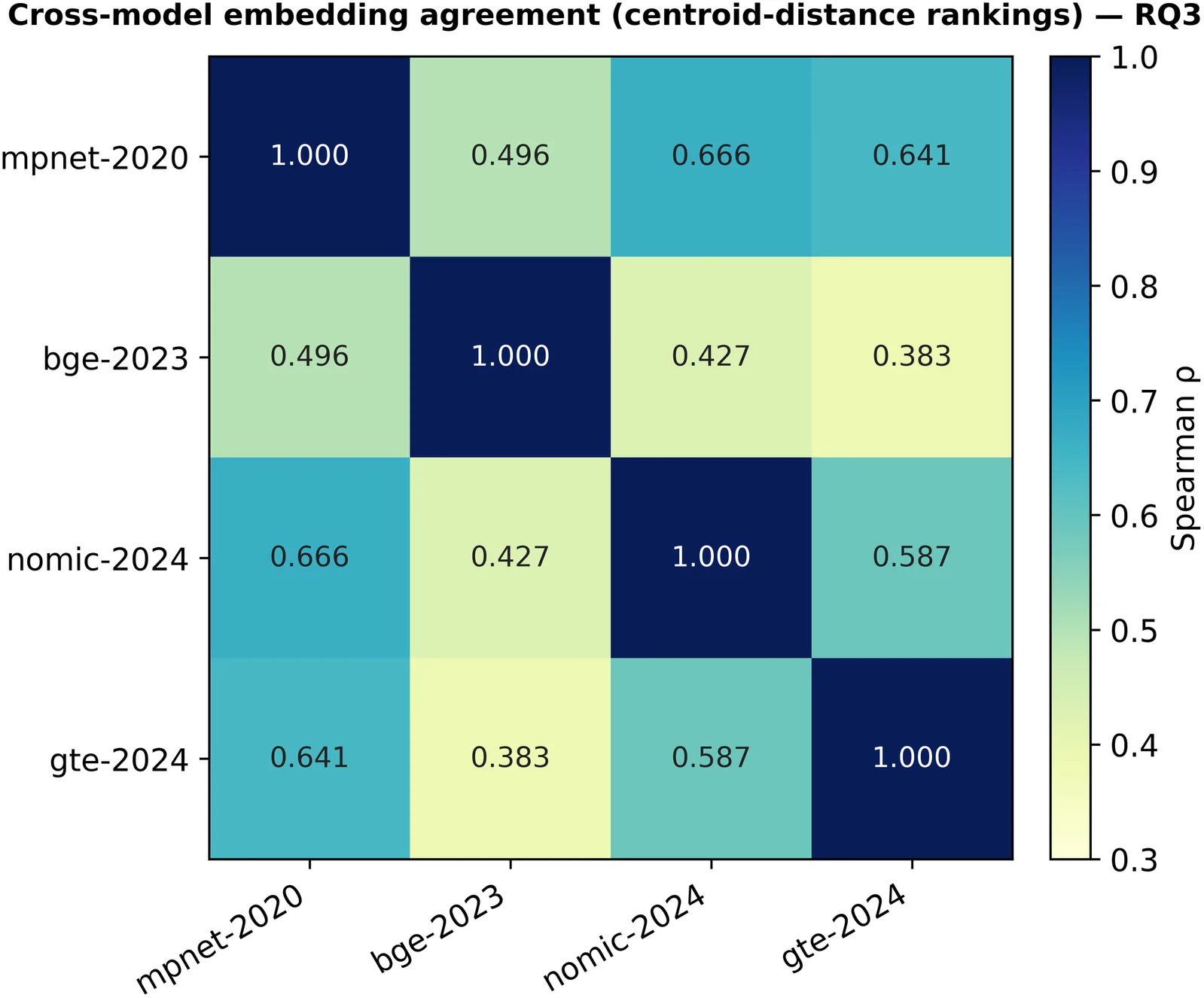
Cross-model embedding consistency. Pairwise Spearman ρ of centroid distance rankings across four embedding models. BGE-2023 shows the weakest agreement with other models.

Correlations range from 0.383 (bge-2023 vs. gte-2024) to 0.666 (mpnet-2020 vs. nomic-2024); the full matrix is given in Table 7 and visualized in Fig 5. The mean per-record percentile standard deviation is approximately 15, meaning percentile ranks vary by roughly ±15 points across models. This moderate consistency suggests that roughly half the variance in semantic position rankings is model-specific, reinforcing the importance of multi-model validation.

**Table 7:** Pairwise Spearman ρ of centroid distance rankings.

	mpnet-2020	bge-2023	nomic-2024	gte-2024
mpnet-2020	1.000	0.496	0.666	0.641
bge-2023	0.496	1.000	0.427	0.383
nomic-2024	0.666	0.427	1.000	0.587
gte-2024	0.641	0.383	0.587	1.000

### 4.8. Effect size summary

**Fig 6:**
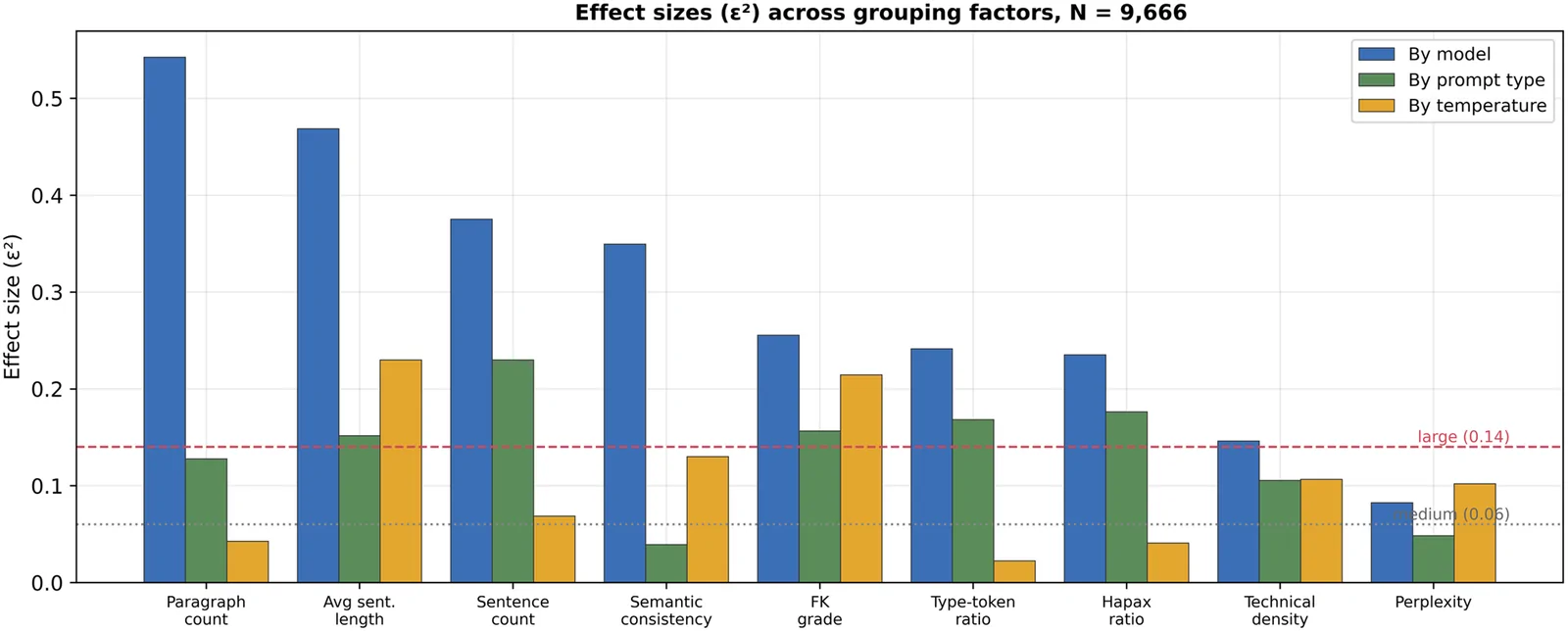
Effect sizes ($\epsilon^{2}$) for key metrics across three grouping factors, N = 9,666. Dashed lines indicate medium (0.06) and large (0.14) thresholds. Model differences dominate in structural metrics; prompt effects are strong across semantic and lexical dimensions.

Of the 39 metrics, 35 are included in cross-group effect-size estimation; the two domain-relevance metrics (used as filter criteria), average syllables per word (collinear with technical-term density), and vocabulary overlap are reported descriptively but omitted from the effect-size tables. Of these 35 metrics, 10 show large model effects ($\epsilon^{2}$ ≥ 0.14), 9 medium (0.06–0.14), and 16 small (< 0.06). For prompt type, 8 metrics show large effects; for temperature, 3. Fig 6 compares these effect sizes across the three grouping factors. The pattern is consistent: structural properties are most sensitive to model choice, lexical and semantic properties are most sensitive to prompt framing, and perplexity-related metrics are most sensitive to temperature.

### 4.9. Centroid robustness

To assess whether the limited calibration set yields stable centroids, we bootstrapped the calibration concepts (1,000 resamples, leave-out resampling, seed 42) and recomputed each domain centroid. The centroids prove highly stable: across all four embedding models and all five domains, the mean cosine similarity between resampled and full-data centroids is 0.990–0.999. A separate baseline probes whether the distance metrics carry genuine domain signal: we compared each output’s distance to its *true* domain centroid against its distance to *wrong-domain* and *label-permuted* centroids. Outputs are closer to their true-domain centroid in 96.8%–98.7% of cases (for all-mpnet, mean true distance 0.530 vs. wrong-domain 0.817 and permuted 0.683). The semantic-position structure is therefore robust to calibration sampling and far stronger than chance.

### 4.10. Threshold sensitivity

The semantic-consistency filter threshold (0.15) is a researcher degree of freedom, so we re-ran the model comparison across thresholds 0.10, 0.15, 0.20, 0.25, and 0.30. Retention is 100% at 0.10–0.15, 91% at 0.20, 64% at 0.25, and 31% at 0.30. The cross-model structural finding is qualitatively stable across this range: paragraph-count $\epsilon^{2}$ is 0.54 (0.10–0.15), 0.48 (0.20), 0.32 (0.25), and remains large at 0.16 even at the most aggressive 0.30 threshold; semantic-consistency $\epsilon^{2}$ follows the same monotone pattern. The primary threshold lies in a flat, high-retention region, and the conclusions do not depend on its precise value.

### 4.11. Embedding architecture and dimensionality

To test whether cross-model agreement is an artifact of embedding dimensionality or recency, we grouped the six embedding-model pairs by shared output dimensionality (768 vs. 1024) and by release year. Agreement is essentially unrelated to shared dimensionality (same-dimension mean ρ = 0.52 vs. different-dimension 0.54) and only weakly related to recency (same-year ρ = 0.59 vs. different-year 0.52). The moderate agreement therefore reflects genuine, architecture-spanning variation in semantic-position estimates rather than a dimensionality artifact.

### 4.12. Manual grammar audit

Because the automated grammar tool was unusable (Section 3.2.1), we manually audited a reproducible stratified random sample of 200 outputs (seed 42; approximately 28–29 per model; balanced across domains and prompt types; 169,266 words), reading each in full and counting only clear prose grammatical errors. We found 9 errors total, with 191/200 (95.5%) outputs error-free and an overall rate of approximately 0.006 errors per 100 words; errors were minor and localized (an article error, a comma splice) and concentrated in GPT-4.1 and the quantized Gemma model. Full per-model results are in Table D of S1 File. This confirms that excluding the automated metric does not admit ungrammatical text.

## 5. Discussion

### 5.1. Summary of findings

Our analysis reveals that LLM-generated scientific concept descriptions are coherent (99.2% pass rate), domain-relevant (99.9%), and exhibit graduate-level readability. The study’s central contribution is the multi-dimensional characterization framework and its three findings (RQ1–RQ3); the bootstrap, baseline, threshold, and audit analyses are supporting validations of those findings. Addressing our three research questions: (RQ1) Models differ most in structural properties—how they organize text—with effect sizes several-fold to an order of magnitude larger than for semantic position (conditional on the semantic-position metrics and calibration we adopt). (RQ2) Prompt framing has substantial effects, with discovery framing producing more academic text closer to known concepts and direct invention pushing further away. Temperature effects are monotonic and replicate across two independent full-range models. (RQ3) Cross-model embedding consistency is moderate (ρ = 0.38–0.67), with approximately half the variance in semantic rankings being model-specific.

### 5.2. Implications

Three implications emerge. First, under the semantic-position operationalization and calibration adopted here, prior work focusing exclusively on semantic distance was measuring a relatively small part of the variation between model outputs—structural differences dominate. Future studies should incorporate structural and lexical dimensions alongside semantic metrics. Second, the moderate embedding model consistency (ρ = 0.38–0.67), which we show is not an artifact of embedding dimensionality, means that claims about semantic position or novelty based on a single embedding model are unreliable; multi-model validation should be a minimum standard. Third, the strong prompt-type effects suggest that apparent differences in model “creativity” may partially reflect prompt sensitivity rather than underlying capability differences.

### 5.3. What the data does not show

These measurements do not demonstrate that any LLM generates *novel* or *creative* scientific concepts. Semantic distance from known concepts is influenced by writing style, vocabulary choice, and text organization—all of which vary substantially across conditions—and cannot be interpreted as novelty without additional validation such as expert review or literature search. The fact that outputs occupy high percentiles in calibration distributions is equally consistent with genuine conceptual innovation, stylistic divergence, or statistical artifacts of comparing long generated text to compact reference descriptions.

### 5.4. Limitations

**Unbalanced temperature design:** While two models (GPT-4.1 and the open-weight Gemma-3-27B) now span the full three-temperature range and replicate the temperature trends (Sections 4.5–4.5.1), the pooled temperature analysis across all seven models remains unbalanced because the Claude and reasoning models do not span the same range. Future work should use a model set that all supports the same temperature range.

**Grammar metric exclusion:** The planned three-metric coherence filter was reduced to two metrics due to LanguageTool failures. A manual audit of 200 outputs (Section 4.12) confirms that the generations are highly grammatical, mitigating this concern, but automated grammaticality tooling robust to markdown and technical notation would allow full-corpus screening in future work.

**Calibration set size:** Thirty-one calibration concepts per domain provide a limited basis for centroid estimation in 768–1024 dimensional space. Centroid stability is now formally assessed by bootstrap resampling (Section 4.9) and is high, but a larger and more diverse calibration set would further tighten distance estimates.

**Threshold sensitivity:** The semantic consistency threshold was lowered from the initially planned 0.30 to 0.15 based on pilot analysis. We now report results across thresholds 0.10–0.30 (Section 4.10) and find the conclusions stable; nonetheless, the choice represents a researcher degree of freedom that future work should pre-register where possible.

**Perplexity reference model:** GPT-2 serves as a fixed, neutral reference for relative coherence screening. Because it predates and is far smaller than the evaluated systems, the absolute perplexity values partly reflect model mismatch rather than output fluency alone; they are therefore used only comparatively and with a permissive threshold (Section 3.5).

**Text preprocessing:** Sentence splitting used regex approaches that may over-split on markdown formatting and bullet points. If models produce systematically different formatting (e.g., more lists vs. prose), this introduces confounds in sentence-level metrics. Models with very high paragraph counts (GPT-5.5 at a median of 123 paragraphs, Claude Opus at 49) may use more structured formatting than compact models (o4-mini at 8).

## 6. Conclusion

We have presented a multi-dimensional characterization of 9,666 LLM-generated scientific concept descriptions from seven models—including a frontier reasoning model and an open-weight model—across five domains, measuring 39 metrics across five dimensions, with post-hoc pairwise comparisons, bootstrap centroid-stability and shuffled-domain baselines, a threshold-sensitivity sweep, and a within-model temperature analysis confirming systematic and robust differences. Our key findings are: (1) structural variation between models dominates semantic variation by several-fold to an order of magnitude in effect size—conditional on our semantic-position operationalization and calibration framework—with all pairwise model differences significant for the top structural metrics; (2) prompt framing systematically shifts both structural and semantic properties, with discovery framing producing outputs closer to known concepts; (3) temperature effects are monotonic and powerful, particularly on perplexity, and replicate across two independent full-range models (GPT-4.1 and Gemma); and (4) semantic position measurements are moderately model-dependent (ρ = 0.38–0.67) in a way not explained by embedding dimensionality. By separating measurement from interpretation, we provide a rigorous empirical foundation for future research on LLM conceptual generation.

## Supporting information

S1 File Supporting information. Contains, as internally labelled sections, the full prompt texts for all three prompt types (Appendix A); complete definitions of the 39 continuous metrics across the five characterization dimensions; centroid bootstrap stability results; the shuffled-domain (label-permutation) baseline; the manual grammar audit by model; the threshold sensitivity analysis; within-model temperature correlations for GPT-4.1; and embedding agreement by dimensionality and release year.
(PDF)
