# Supplementary material for: Characterizing LLM scientific concept generation: A multi-dimensional measurement study
Source: PLoS One. 2026 Sep 15;21(9):e0357892. doi: 10.1371/journal.pone.0357892 (PMC13577575; doi:10.1371/journal.pone.0357892)
Supplement: Supplementary file 1 [file pone.0357892.s001.pdf]

Supporting Information

Characterizing LLM Scientific Concept Generation: A Multi-Dimensional Measurement Study

Jalil Ahmadpour

Appendix A. Full prompt texts

Three prompt types were used across five domains (15 prompts total). Each model received the exact text below for the corresponding domain × prompt type combination. Fifty independent generations were produced per condition.

Direct invention prompts

Physics: Invent a completely new hypothetical subatomic particle that is NOT in the Standard Model and has NOT been proposed before in physics literature. Provide: 1) A novel name (not used in physics) 2) Three unique properties that distinguish it from ALL known particles (electrons, quarks, neutrinos, Higgs, etc.) 3) How it would interact with known particles 4) A potential experimental signature for detecting it. Be maximally creative and original — avoid anything resembling existing particles, proposed particles, or established theories. Do not combine or modify existing concepts.
Biology: Invent a completely new biological mechanism for cellular communication that does NOT exist in any known organism. Provide: 1) A novel name for this mechanism 2) Detailed molecular description of how it works 3) What signals it transmits and how 4) Why it would provide advantages over existing mechanisms. Avoid anything resembling existing mechanisms: chemical signaling (hormones, neurotransmitters), electrical signaling, gap junctions, exosomes, quorum sensing, etc. Create something genuinely unprecedented.
Mathematics: Invent a completely new mathematical operation or structure that does NOT exist in any branch of mathematics. Provide: 1) A novel name and symbol for the operation/structure 2) A rigorous formal definition 3) Key properties and theorems that would hold 4) One or two worked examples. Create something genuinely unprecedented — not a combination or variation of existing operations (addition, multiplication, exponentiation, integration, etc.) or structures (groups, rings, manifolds, etc.).
Philosophy: Create a new philosophical concept about the nature of consciousness or mind that has NOT been proposed in philosophy. Provide: 1) A novel name for this concept/position 2) Clear definition and key claims 3) Main arguments supporting the position 4) How it differs from ALL existing positions (dualism, materialism, functionalism, panpsychism, idealism, eliminativism, etc.). Create something genuinely new, not a synthesis or modification of existing views.
Economics: Propose a new economic mechanism or institution that does NOT exist and has NOT been proposed in economics. Provide: 1) A novel name 2) How it would function in detail 3) What problem it would solve or what benefit it would provide 4) How it differs from ALL existing mechanisms (markets, auctions, voting systems, contracts, etc.). Create something genuinely unprecedented, not a modification of existing economic institutions.

Discovery framing prompts

Physics: Describe a hypothetical physics discovery that could be made at a particle accelerator in 2035. This discovery should reveal something fundamentally new about the universe that: 1) Is NOT predicted by any current theory (Standard Model, string

theory, loop quantum gravity, etc.) 2) Would require new physics principles to explain 3) Has specific measurable properties you describe in detail 4) Would have profound implications for our understanding of reality. Describe the discovery as if writing a scientific announcement, including the key experimental findings and theoretical implications.
Biology: Describe a hypothetical biological discovery that could be made in 2035 that reveals a fundamentally new aspect of life. This discovery should: 1) Involve a new type of biological entity, process, or organization not currently known 2) Challenge or extend current understanding of how life works 3) Have specific measurable properties you describe in detail 4) NOT be an extension of known biology (new species, new genes, new proteins in known families). Describe as if writing a scientific paper abstract with key findings and implications.
Mathematics: Describe a hypothetical mathematical discovery that could be made by a mathematician in 2035. This discovery should: 1) Reveal a new mathematical truth or structure not currently known or conjectured 2) Have profound implications for mathematics 3) Be describable with some precision (not vague) 4) NOT be the solution to an existing open problem. Describe as if announcing the discovery, including the key insight and its significance.
Philosophy: Describe a hypothetical philosophical breakthrough that could emerge from a philosopher in 2035. This breakthrough should: 1) Offer a genuinely new insight about a fundamental philosophical question 2) NOT be derivable from existing philosophical positions 3) Have clear implications that you describe 4) Present a new way of thinking, not just new arguments for old positions. Describe as if summarizing a groundbreaking philosophy paper.
Economics: Describe a hypothetical economic discovery that could emerge from research in 2035. This discovery should: 1) Reveal something fundamentally new about economic behavior or systems 2) NOT be predictable from current economic theory 3) Have specific empirical content you describe 4) Challenge existing economic assumptions. Describe as if summarizing findings from groundbreaking economic research.

Theoretical framework prompts

Physics: Propose a new theoretical framework in physics that transcends or unifies existing approaches. This framework should: 1) Have a novel name and core principle not found in existing physics 2) Address a fundamental question (nature of space, time, matter, or forces) in a genuinely new way 3) Make specific predictions that differ from all existing theories 4) NOT be a combination or extension of existing frameworks (quantum mechanics, relativity, field theory, etc.). Describe the mathematical or conceptual foundation and key implications.
Biology: Propose a new theoretical framework in biology that transcends current understanding. This framework should: 1) Have a novel name and organizing principle 2) Explain some aspect of life in a fundamentally new way 3) Make predictions that differ from current biological theory 4) NOT be an extension of evolution, genetics, systems biology, or other established frameworks. Describe the core concepts and how they would reshape biological understanding.
Mathematics: Propose a new branch or theoretical framework in mathematics that transcends existing approaches. This framework should: 1) Have a novel name and foundational concepts 2) Study mathematical objects or relationships not currently formalized 3) Connect to but go beyond existing mathematics 4) NOT be a direct extension of existing fields (topology, algebra, analysis, etc.). Describe the foundational definitions and key theorems of this new framework.
Philosophy: Propose a new philosophical framework that transcends existing approaches to a major philosophical problem (consciousness, knowledge, ethics,

existence, etc.). This framework should: 1) Have a novel name and core principle 2) Dissolve or reframe the problem in a genuinely new way 3) NOT be a synthesis of existing positions 4) Have clear implications for how we should think about the issue. Describe the framework's foundations and key insights.
Economics: Propose a new theoretical framework in economics that transcends existing approaches. This framework should: 1) Have a novel name and core principles (3–4) 2) Explain economic phenomena in a fundamentally new way 3) NOT be a synthesis of existing frameworks (neoclassical, Keynesian, behavioral, Marxist, Austrian, etc.) 4) Make predictions that differ from current economic theory. Describe the framework's foundations and key implications.

Generation parameters

Parameter	Value
Repetitions per condition	50
System prompt	None
Original generation period	January 3–6, 2026
Added-model generation period	June 2026 (GPT-5.5, Gemma-3-27B)
Total raw outputs	9,770

Table A. Complete metric definitions

The semantic-position dimension (D5) comprises, for each of the four embedding models, five continuous metrics (centroid cosine distance, centroid Euclidean distance, nearest-known distance, average distance to known, and percentile in known) — 20 per-model metrics — plus a per-model binary in-distribution flag, together with three cross-model consistency metrics (percentile mean, standard deviation, and range). The 20 per-model continuous metrics + 3 cross-model metrics + the 16 metrics of D1–D4 (2 + 2 + 6 + 6) give the **39 continuous metrics** analyzed throughout; the four binary in-distribution flags (one per embedding model) are reported descriptively. Per-model D5 metrics are omitted from the table below for brevity but follow identical definitions across all four models.

Dim.	Metric	Definition	Interpretation
D1	Perplexity	GPT-2 cross-entropy (exponentiated)	Lower = more coherent
D1	Semantic consistency	Mean pairwise cosine similarity of sentence embeddings	Higher = more internally consistent
D2	Keyword density	Domain-specific terms / total words × 100	Higher = more domain-specific
D2	Core keyword hits	Count of high-precision domain terms	Higher = more domain-specific
D3	Type-token ratio	Unique words / total words	Higher = more lexically diverse
D3	OOV rate	Non-dictionary words / total words	Higher = more technical vocabulary
D3	Vocabulary overlap	Shared tokens with calibration / unique words	Higher = more overlap with known concepts
D3	Technical term density	Words with 3+ syllables / total words	Higher = more complex vocabulary

Dim.	Metric	Definition	Interpretation
D3	Hapax legomena ratio	Words occurring once / unique words	Higher = less repetitive
D3	Avg syllables per word	Mean syllables across all words (under-counts symbolic content)	Higher = more complex vocabulary
D4	Avg sentence length	Mean words per sentence	Higher = more complex syntax
D4	Sentence count	Total number of sentences	Higher = longer output
D4	Paragraph count	Total number of paragraphs	Higher = more structured
D4	Flesch-Kincaid grade	FK readability grade level	Higher = harder to read
D4	Flesch reading ease	FK reading ease score	Lower = harder to read
D4	Definition density	Definitional sentences / total sentences	Higher = more definitional style
D5	Centroid dist. (cosine)	Cosine distance to domain centroid embedding	Higher = further from domain center
D5	Centroid dist. (Euclidean)	Euclidean distance to domain centroid	Higher = further from domain center
D5	Nearest known dist.	Cosine distance to closest calibration concept	Higher = less similar to any known concept
D5	Avg dist. to known	Mean cosine distance to all calibration concepts	Higher = less similar overall
D5	Percentile in known	Rank position in calibration distance distribution	Higher = more distant from known
D5	In-distribution flag	Whether output falls within 2σ of calibration	Boolean
D5	Cross-model pct. mean	Mean percentile rank across 4 embedding models	Aggregate semantic position
D5	Cross-model pct. std	Standard deviation of percentile across models	Higher = less consistent across models
D5	Cross-model pct. range	Max minus min percentile across models	Higher = greater model disagreement

---

Table B. Centroid bootstrap stability

Calibration concepts were resampled 1,000 times (leave-out resampling, seed 42) and domain centroids recomputed. Values are the cosine similarity between resampled and full-data centroids (range of per-domain means across the five domains).

Embedding model	Cosine similarity to full-data centroid (min–max across domains)
mpnet-2020	0.990 – 0.993
bge-2023	0.997 – 0.998
nomic-2024	0.998 – 0.999
gte-2024	0.997 – 0.998

Mean per-generation percentile drift under resampling was small (e.g., mpnet-2020 mean ≈ 3.9 percentile points, P95 ≈ 12.4).

Table C. Shuffled-domain (label-permutation) baseline

For each embedding model, mean cosine distance of outputs to their **true** domain centroid versus **wrong-domain** and **label-permuted** centroids, with the percentage of outputs closer to the true-domain centroid.

Embedding model	True-domain	Wrong-domain	Permuted	Closer to true (%)
mpnet-2020	0.530	0.817	0.683	96.8
bge-2023	0.222	0.340	0.260	97.6
nomic-2024	0.207	0.288	0.237	97.3
gte-2024	0.278	0.435	0.346	98.7

Table D. Manual grammar audit (per model)

A stratified random sample of 200 outputs (seed 42; balanced across the seven models, five domains, and three prompt types; 169,266 words total) was read in full; only clear prose grammatical errors were counted.

Model	Outputs	Total errors	Mean words/output	Errors per 100 words	Error-free outputs (%)
Claude Opus 4.5	29	0	1,026	0.000	100.0
Claude Sonnet 4.5	29	1	596	0.005	96.6
GPT-5.2	28	0	644	0.000	100.0
GPT-5.5	28	0	1,652	0.000	100.0
o4-mini	28	0	576	0.000	100.0
GPT-4.1	29	5	538	0.026	82.8
Gemma-3-27B	29	3	904	0.011	89.7
**Overall**	**200**	**9**	**846**	**0.006**	**95.5**

The nine errors were minor and localized (article errors, comma splices, a dangling modifier, a subject–verb agreement slip). Four of the seven models had no errors.

Table E. Threshold sensitivity

The model comparison was re-run across semantic-consistency filter thresholds 0.10–0.30. Effect sizes are epsilon-squared by model.

Threshold	Retention (%)	Paragraph-count ϵ^2	Semantic-consistency ϵ^2
0.10	100.0	0.542	0.349

Threshold	Retention (%)	Paragraph-count ϵ^2	Semantic-consistency ϵ^2
0.15 (primary)	100.0	0.542	0.349
0.20	91.1	0.476	0.287
0.25	63.9	0.320	0.178
0.30	30.6	0.160	0.125

The cross-model structural finding remains large ($\epsilon^2 \geq 0.14$) across the entire range, including the most aggressive 0.30 threshold that discards 69% of outputs.

Table F. Within-model temperature correlations (GPT-4.1, n = 2,219)

Spearman correlations between temperature and each metric, computed within GPT-4.1 to isolate temperature from model identity.

Metric	Spearman ρ with temperature
Perplexity	+0.769
Hapax legomena ratio	+0.519
Type-token ratio	+0.438
Flesch-Kincaid grade	+0.373
OOV rate	+0.242
Average sentence length	+0.266
Technical term density	+0.200
Flesch reading ease	−0.346
Semantic consistency	−0.174

The open-weight Gemma-3-27B model (full temperature range) reproduces the same directional pattern from t=0.7 to t=1.3: perplexity 31.2 → 33.7 → 35.5, type-token ratio 0.464 → 0.471 → 0.476, technical-term density 0.346 → 0.348 → 0.353, average sentence length 17.5 → 17.8 → 18.0.

Table G. Embedding agreement by dimensionality and release year

Pairwise Spearman ρ of centroid-distance rankings, annotated by whether the pair shares output dimensionality and release year.

Pair	ρ	Same dimensionality?	Same year?
mpnet-2020 vs nomic-2024	0.666	Yes (768)	No
mpnet-2020 vs gte-2024	0.641	No	No
nomic-2024 vs gte-2024	0.587	No	Yes (2024)
mpnet-2020 vs bge-2023	0.496	No	No
bge-2023 vs nomic-2024	0.427	No	No
bge-2023 vs gte-2024	0.383	Yes (1024)	No

Group means: same-dimensionality pairs $\rho = 0.52$ vs. different-dimensionality 0.54; same-year pairs $\rho = 0.59$ vs. different-year 0.52. Agreement is not explained by shared dimensionality and is only weakly related to recency.
